# Quantitative validation of the “coastline” heuristic for café-au-lait macule borders in neurofibromatosis type 1 and McCune-Albright syndrome

**DOI:** 10.1016/j.jaad.2026.08.007

**Published:** 2026-08-07

**Authors:** Maya DiFrischia, Ping Hu, Rebekah L. Waikel, Benjamin D. Solomon, Dat Duong

**Affiliations:** Section on Emerging Analytics, Medical Genetics Branch, National Human Genome Research Institute, Bethesda, Maryland.

**Keywords:** artificial intelligence, café-au-lait macules, café-au-lait spots, fractal analysis, McCune-Albright syndrome, neurofibromatosis type 1

Many genetic conditions involve distinctive physical features whose recognition enables efficient diagnosis and disease management. Artificial intelligence (AI) methods can help clinicians identify these features by facilitating accurate segmentation and classification. We implemented AI methods to evaluate a classic “textbook lesson” for clinicians. This lesson teaches that the borders of café-au-lait macules (CALMs) in Neurofibromatosis type 1 (NF1) are smooth like the “coast of California”, while CALMs in McCune-Albright syndrome (MA) have jagged borders like the “coast of Maine.”^1^ We verified this lesson by quantifying macule border irregularity with AI-enabled fractal dimension analysis.^2^

We assembled 311 images of CALMs (NF1:228; MA:83) through searches of public databases. Diagnoses were verified by board-certified clinicians, though caution is warranted due to genetic heterogeneity in clinically-diagnosed individuals.^3^ To accurately isolate the CALM borders, we created a fine-tuned model based on Segment Anything (SAM), and trained it on a set of manually segmented CALMs (NF1:31; MA:23).^4^ More details regarding the methods and findings are described in the Supplementary materials, available via Mendeley at https://data.mendeley.com/datasets/9hvxg4wdfx/1.

This method significantly improved segmentation predictions for the remainder of the dataset, though individual results varied (Supplementary Fig 2, available via Mendeley at https://data.mendeley.com/datasets/9hvxg4wdfx/1). Examining a random 60 image subset, fine-tuned SAM in automatic instance segmentation improved average Hausdorff distance from 41.51 pixels (STD: 35.44 pixels) to 2.40 pixels (STD: 6.60 pixels) (*P* = 7.0 × 10^−12^). The fine-tuned model was also compared to baseline SAM with bounding-box prompts, which performed similarly; ultimately, the fine-tuned model provided superior edge segmentation (Supplementary Fig 3, available via Mendeley at https://data.mendeley.com/datasets/9hvxg4wdfx/1). In addition, our fine-tuned model demonstrates some generalizability for the segmentation of other genodermatoses, though condition-specific fine-tuning would likely further improve outputs (Supplementary Fig 4, available via Mendeley at https://data.mendeley.com/datasets/9hvxg4wdfx/1). We manually reviewed the binary segmentations and selected accurate results for fractal analysis (NF1:60; MA:25) (Supplementary Fig 5, available via Mendeley at https://data.mendeley.com/datasets/9hvxg4wdfx/1).

To provide standardization, segmentation outputs were cropped with fifty-pixel paddings from the CALM boundary edges, which preserved edge morphology while allowing consistent fractal dimension analysis despite source image variability. The states were imported from R package “state_boundaries_wgs84” and edited to only include the coastlines. Multifractal calculations were completed using the ImageJ version 2.16.0/1.54p plugin FracLac version 2.5 for the selected images and state coastlines in order to quantify border irregularity.^5^

Multifractal analysis revealed a significant difference between the fractal dimensions of NF1 versus MA CALMs (*P* = 3.1 × 10^−1^) (Fig 1). The average dimension of NF1 CALMs was 1.115 ± 0.051, not significantly different from the dimension of the California coast, 1.100 (*P* = .442). Likewise, the average dimension of MA CALMs was 1.248 ± 0.060, not significantly different from the Maine coast 1.267 (*P* = .133).

Additionally, we trained an image classifier using Low Rank Adaptation (LoRA), a fine-tuning technique that uses relatively few images. Updating only 1.8% of the parameters, this fine-tuning method yielded a classifier accuracy of 94%. Of the misclassified CALMs, 60% had fractal dimensions outside of the expected range for their diagnostic group (Supplementary Table, available via Mendeley at https://data.mendeley.com/datasets/9hvxg4wdfx/1).

In summary, our results mathematically support the classic textbook lesson about distinguishing CALMs. Our methods also demonstrate how relatively small, heterogeneous skin image datasets can yield significant and meaningful results via AI.

## Figures and Tables

**Fig 1.**
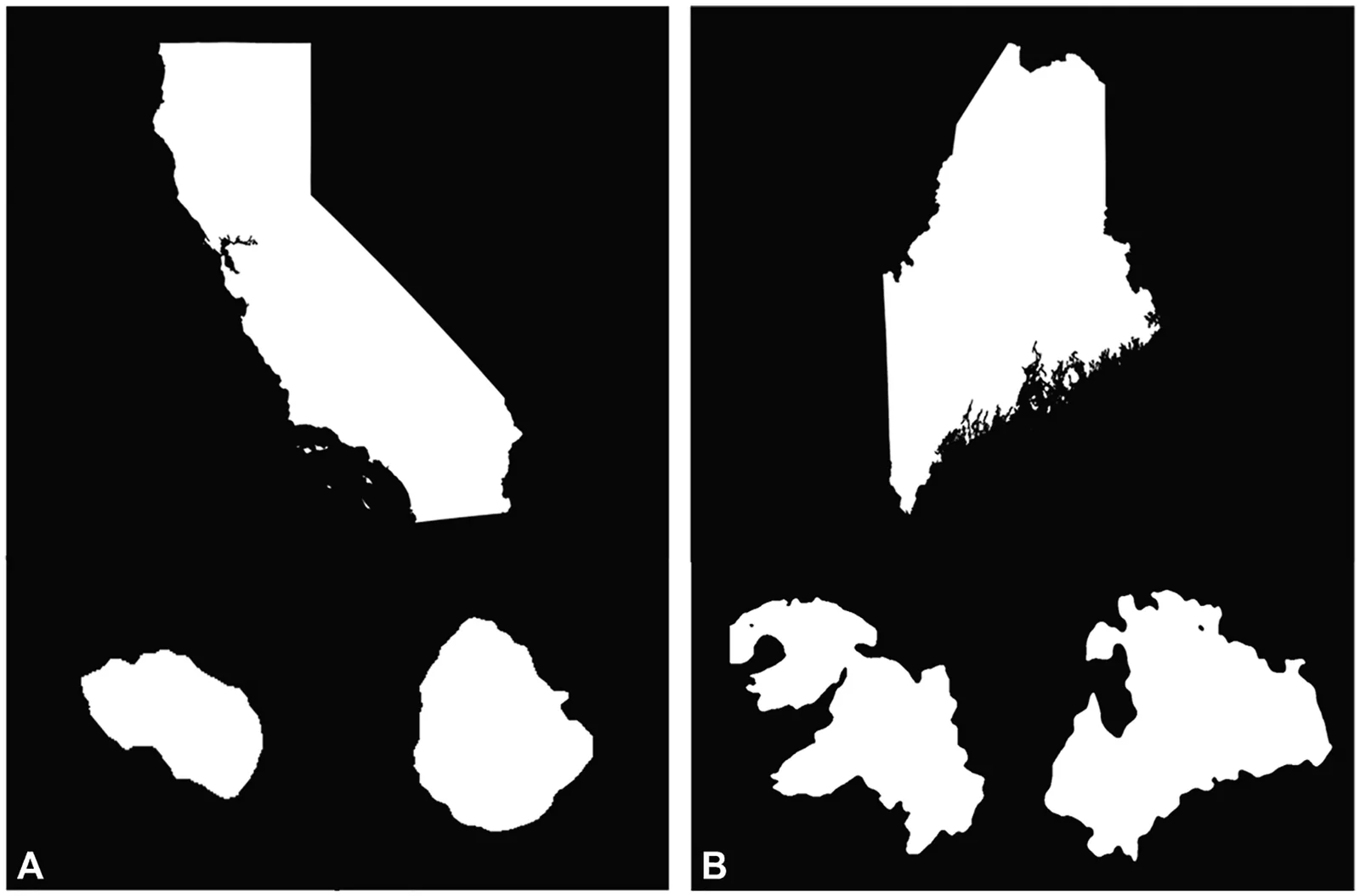
**A,** Contour of the state of California accompanied below by exemplary neurofibromatosis type 1 (NF1) binarized café-au-lait macules (multifractal dimensions of 1.12 and 1.09, respectively). **B,** Contour of the state of Maine accompanied below by exemplary McCune-Albright syndrome (MA) binarized café-au-lait macules (multifractal dimensions of 1.21 and 1.26, respectively). Note that only the state coastlines were considered in fractal dimension calculations. Therefore, we examined the left border of California (1.10) and the right lower border of Maine (1.267) to find fractal dimensions.

## Key Message:

Our results mathematically *support* the classic textbook lesson about distinguishing café-au-lait macules, and demonstrate how relatively small, heterogeneous skin image datasets can yield significant and meaningful results via AI.
